# Supplementary material for: Methodological establishment and diagnostic value of a multiplex fluorescent PCR assay for the detection of three fastidious respiratory pathogens
Source: PLoS One. 2025 Jul 31;20(7):e0328651. doi: 10.1371/journal.pone.0328651 (PMC12312904; doi:10.1371/journal.pone.0328651)
Supplement: S1 Table — This table lists the PCR reagents, primer suppliers, DNA extraction kits, and PCR instruments. (DOCX) [file pone.0328651.s001.docx]

**Supplementary table 1: Main Reagents and Instruments**

| **Reagents and Instruments** | **Supplier** |
| --- | --- |
| SLAN-96P fully automated medical PCR analysis system | Shanghai Hongshi Medical Technology Co., Ltd. |
| VITEK2Compact fully automated bacterial identification and drug sensitivity analysis system | bioMérieux (France) |
| Sputum digest | Oxoid (UK) |
| Bacterial genome DNA extraction kit | Tengen Biochemistry Science and Technology (Beijing) Co., Ltd. |
| 2× Accurate TaqHS Probe Premix (UNG Plus) | EcoRes Bioengineering (Changsha, Hunan, China) |
