# Supplementary material for: Methodological establishment and diagnostic value of a multiplex fluorescent PCR assay for the detection of three fastidious respiratory pathogens
Source: PLoS One. 2025 Jul 31;20(7):e0328651. doi: 10.1371/journal.pone.0328651 (PMC12312904; doi:10.1371/journal.pone.0328651)
Supplement: S2 File — (DOCX) [file pone.0328651.s002.docx]

**tNGS Workflow on the Nanopore Platform**

NTS testing: BALF samples (>5mL, placed in sterile sputum containers) are collected and immediately transported to a commercial laboratory (Hangzhou Dean Medical Laboratory (Hangzhou, China) using dry ice for testing.

Supplementary Table 2.1: Instruments and Equipment Used

| Instrument name | Model | Manufacturer and Producer |
| --- | --- | --- |
| Desktop refrigerated centrifuge | BY-R320 | Baiyang, Beijing |
| Vortex Mixers | QL-901 | Qilinbeire, Haimen |
| High speed centrifuge | BY-G1200 | Baiyang, Beijing |
| Tissue grinding homogenizer | TL2020S | Dinghaoyuan, Tianjing |
| Fluorescence quantizer | qubit 4.0 | Thermo Fisher |
| Constant temperature water bath | DKT-200 | Miou, Hangzhou |
| PCR instrument | Hema9600 | Heima, Zhuhai |
| Nanopore sequencer | GridION MK1 | Oxford Nanoporous Technology |

**1. Sample Preprocessing**

Liquefaction of alveolar lavage fluid (only for viscous samples), add a certain amount of liquefied fluid and 20% by volume 1:1 μ L XC, shake and mix well, shake at 1000 rpm on a constant temperature mixer for 1-3 minutes, incubate at 37 ℃ for 10 minutes (if the liquefaction is still viscous, increase the amount of liquefaction liquid appropriately for secondary liquefaction), take 1mL (the maximum volume of less than 1mL) and add it to a 1.5mL sterile centrifuge tube, centrifuge at 12000 rpm for 2 minutes, and discard 950 μ L supernatant (residual liquid approximately 30-40 μ L) . Add 200 μ L cracking solution LS, shake or blow with a gun head to suspend and disperse the precipitate, and react at 37 ℃ for 10 minutes. Add 100mg of glass beads and shake the wall breaker at 2000rpm for 8 minutes (pausing for 30 seconds every 1 minute) at high speed.

**2. Nucleic acid extraction**

Add 400 μ L cracking liquid LE, 20 μ L Proteinase K, blow and mix well, and absorb about 600 after brief centrifugation μ Add L supernatant to the prepared 96-well deep well plate in the 1st/7th row of holes, and place the 96-well plate and magnetic sleeve into the Hema nucleic acid automatic extractor according to the requirements of the instrument manual, and automatically run according to the set program. After the program is completed, transfer the nucleic acid solution from the 6/12 well to a 1.5 mL EP tube and measure the DNA concentration using a Qubit 3.0 fluorescence quantitative analyzer.

**3. Library construction**

**3.1 Targeted amplification**

Prepare a reverse transcription reaction mixture in a 0.2 mL PCR centrifuge tube according to the ratio in Supplementary Table 2.2 (the reaction solution needs to be prepared and packaged on an ice box, and the ice box needs to be kept at low temperature), mix it gently, centrifuge briefly, and place it in a PCR machine for reverse transcription reaction. The reaction conditions are as follows: 37 ℃ for 15 minutes, 85 ℃ for 5 seconds, 4 ℃ hold。

Supplementary Table 2.2: Reverse Transcription Reaction Mixture

| Component | Per sample/volume（μL) |
| --- | --- |
| RNA | 8 |
| Reverse transcription reaction fluid | 2 |
| Total | 10 |

Prepare the required amount of PCR reaction mixture in a new centrifuge tube as shown in Supplementary Table 2.3, and perform the PCR reaction according to the reaction conditions shown in Supplementary Table 2.4 after completion.

Supplementary Table 2.3: PCR Reaction System

| Component | Per sample/volume（μL) |
| --- | --- |
| Reverse transcript product | 4μl |
| PCR amplification reaction solution | 36μl |
| Total | 40 μl |

Supplementary Table 2.4: PCR Reaction Procedure

| Temperature | Time | Cycle |
| --- | --- | --- |
| 95℃ | 2 min | 1 |
| 95℃ | 15 s | 35 |
| 60℃ | 2min 15 s |  |
| 72℃ | 5 min | 1 |
| 4℃ | Hold | 1 |

After the reaction is completed, add 1.5X volume of magnetic beads for purification, blow and mix well, and let stand for 5 minutes; Transfer to a magnetic rack, wait for the solution to clear (about 2 minutes), discard the supernatant, and use 150 μ Wash twice with 70% -80% ethanol (do not disturb the magnetic beads), immediately remove and place the remaining ethanol on a magnetic rack with 10 μ L Pipette absorbs the solution, leaves it to dry for 2min, and finally washes it with 25 µ L ddH2O water at room temperature for 5min. Place it on the magnetic rack, and after the solution is clarified (about 2min), suck out the eluent for standby and measure the concentration.

**3.2 DNA fragment end repair and purification**

Prepare the final reaction solution according to Supplementary Table 2.5 in a 0.2ml PCR tube, gently stir and mix, 20 ℃ for 5 minutes, 65 ℃ for 5 minutes, 4 ℃ hold

Supplementary Table 2.5: DNA Fragment End Repair and Purification System

| Reagent | Volume |
| --- | --- |
| H_2_O | Calculate the volume of added DNA and replenish it with water to 12.5ul |
| DNA |  |
| Terminal modifying Buffer | 1.75µl |
| Terminal modifying enzyme | 0.75µl |
| Total | 1. µl |

Prepare reaction solution according to Supplementary Table 2.6, gently stir and mix, 20 ℃ for 20 minutes, 65 ℃ for 10 minutes, 4 ℃ hold；

Supplementary Table 2.6: Barcode Connection Reaction System

| Reagent | Volume |
| --- | --- |
| H2O | 2.5 µl |
| erminal modification products | 5 µl |
| Native Barcode | 2.5 µl |
| Connect the reaction liquid | 10 µl |
| Total | 20 µl |

After connecting Barcode, mix all the samples into one tube. Add 1.5 * magnetic beads to the sample tube, blow and mix well, and let it stand at room temperature for 10 minutes (gently stir and mix 2-3 times with your fingers); After the rest is completed, immediately detach and transfer to a magnetic rack. After the solution clarifies (about 5 minutes), discard the supernatant; Remove the sample tube from the magnetic holder and clean it twice with 1.5 * SFB. After each addition of SFB, it should be thoroughly mixed with the magnetic beads, then immediately separated and placed on the magnetic holder. After clarification, discard the liquid. Add 100 µ l of 80% ethanol (do not disturb the magnetic beads), immediately dissociate, remove the remaining ethanol, and dry for 30 seconds; Add 35 µ l of water, blow and mix well, let stand at room temperature for 5 minutes, transfer immediately to a magnetic rack, wait for the solution to clear, transfer for backup, and measure the concentration.

**3.3 Adapter Mix (AMX) Connection Reaction and Purification**

Prepare the reaction solution according to Supplementary Table 2.7, gently stir and mix, and react at 20 ℃ for 20 minutes；

Supplementary Table 2.7: Adapter Connection Reaction System

| Reagent | Volume |
| --- | --- |
| Pooled barcoded sample | 30 µl |
| Adapter Mix II (AMII) | 5 µl |
| Ligation buffer | 10 µl |
| ligase | 5 µl |
| Total | 50 µl |

Add 75 to the connecting product after the reaction is completed μ Purify with L magnetic beads, blow and mix well, let stand for 10 minutes, and use 125 μ Clean the L SFB twice, mix it thoroughly with the magnetic beads after each addition of SFB, and then place it on a magnetic rack after instant separation. After clarification, discard the liquid. Immediately after separation, place it on a magnetic rack and apply 10% residual SFB μ L Pipette shall be sucked clean and dried after standing for 30s; Join 12 μ L EB, blow and mix well, let stand for 5 minutes, transfer to a magnetic rack after instant separation, and wait for the solution to clear before measuring the concentration and backup.

**4. Machine sequencing**

Melt Sequencing Buffer (SQB), Loading Beads (LB), Flush Tether (FLT), and a tube of Flush buffer (FB) at room temperature. Prepare priming mix in a 1.5 mL centrifuge tube: Take 1 FB and add 30 μ L FLT, shake well and centrifuge briefly. Open the cover of MinION Mk1B and load the chip. Slide the sampling port cover clockwise, open the chip Priming port, set the 1mL Pipette to 180 µ L, insert the suction head of the Pipette into the Priming port, slowly rotate the rotary wheel counterclockwise to make the dial display 200 µ L, or until a small amount of buffer can be seen entering the suction head, and then draw 800 μ The priming mix of L is slowly injected into the flow cell from the Priming port to avoid bubbles, and it is allowed to stand at room temperature for 5 min. Fully mix the loading beads (LB) with a Pipette. In the new EP tube, prepare the sample library as follows: 37.5 µ L Sequencing Buffer (SQB), 25.5 µ L LB, 40-80 ng DNA library, fill with water to 75 µ L, and perform sequencing on the machine.

1. **Bioinformatics analysis**

The format of the original data file generated by MinION sequencer is fast5. The MinKnow software is used to complete real-time identification and generate the fastq file, and the MinKnow software is used to filter low quality value sequences. The filtered data was subjected to host DNA removal using Minimap2 software (using the human genome reference sequence Hg38). Multi Sequence alignment of sequencing data and determination of pathogenic microorganisms After data filtering and de host DNA sequencing data, Centri figure v1.0.3 is used for multi Sequence alignment with NCBI non redundant nucleic acid data (NT) library.
