# Supplementary material for: Methodological establishment and diagnostic value of a multiplex fluorescent PCR assay for the detection of three fastidious respiratory pathogens
Source: PLoS One. 2025 Jul 31;20(7):e0328651. doi: 10.1371/journal.pone.0328651 (PMC12312904; doi:10.1371/journal.pone.0328651)
Supplement: S3 Table — This includes Ct values, means, standard deviations, and coefficients of variation (CV) for each pathogen. (DOCX) [file pone.0328651.s003.docx]

**Supplementary table 3: Repeatability analysis of TaqMan probe multiplex fluorescent PCR at different concentrations**

| Pathogenic  species | The Ct values of 3 tests | | | Means | S | CV |
| --- | --- | --- | --- | --- | --- | --- |
|  | 1st | 2nd | 3rd |  |  |  |
| SP | 24.50 | 24.48 | 24.49 | 24.49 | 0.00816 | 0.03334 |
|  | 28.81 | 28.76 | 28.82 | 28.80 | 0.02625 | 0.09114 |
|  | 31.49 | 31.59 | 31.65 | 31.58 | 0.06600 | 0.20900 |
| HI | 23.12 | 23.15 | 23.15 | 23.14 | 0.01414 | 0.06112 |
|  | 27.44 | 27.37 | 27.42 | 27.41 | 0.02944 | 0.10740 |
|  | 29.72 | 29.68 | 29.73 | 29.71 | 0.02160 | 0.07271 |
| MC | 27.94 | 27.99 | 28.04 | 27.99 | 0.04082 | 0.14586 |
|  | 31.39 | 31.33 | 31.39 | 31.37 | 0.02828 | 0.09016 |
|  | 34.87 | 34.90 | 34.79 | 34.85 | 0.04643 | 0.13321 |

SP：*Streptococcus pneumoniae* HI：*Haemophilus influenzae* MC：*Moraxella catarrhalis*

S：Standard Deviation CV：Coefficient of Variance
